# Comparing quality of life between patients undergoing trans-areola endoscopic thyroid surgery and trans-oral endoscopic thyroid surgery

**DOI:** 10.1186/s12893-021-01267-3

**Published:** 2021-06-03

**Authors:** Shuang Shen, Xiaochi Hu, Rui Qu, Youming Guo, Libo Luo, Xin Chen

**Affiliations:** Department of Breast & Thyroid Surgery, Third Affiliated Hospital of Zunyi Medical University/First People’s Hospital of Zunyi, Zunyi, 563000 China

**Keywords:** Trans-oral thyroidectomy, Trans-areolar thyroidectomy, Quality of life, Cosmetic satisfaction

## Abstract

**Background:**

Compared with conventional open surgery, endoscopic thyroidectomy via the oral vestibular approach (ETVOA) and endoscopic thyroidectomy via the areola approach (ETAA) avoided scarring of the skin, which may help patients achieve a better quality of life (QOL). However, the benefit of the QOL from this technique has not been adequately investigated, therefore this study compared the QOL outcomes between ETVOA and ETAA.

**Methods:**

131 patients were enrolled in this study. ETAA surgery and ETVOA surgery were performed in 74 patients and 57 patients, respectively. These patients were followed up at 2 weeks, 4 weeks, and 8 weeks, and their QOL was evaluated using a thyroid surgery-specific questionnaire and a short-form health survey (SF-36).

**Results:**

There were no differences in clinical characteristics such as gender, age, body mass index (BMI), and tumor size between the two groups. The volume of intraoperative blood loss, cost of hospitalization, and complications between the two procedures showed no differences. Compared with ETAA, ETVOA has a longer operation time, no drainage, and shorter hospital stay. In the QOL questionnaire, several parameters in ETVOA were better. The satisfaction scores of patients undergoing ETVOA were higher. In addition, the cosmetic satisfaction in patients who received ETOVA was significantly better than that of patients who underwent ETAA. The degree of neck movement disorder in patients with ETVOA was milder. Patients who received ETVOA had higher score on the SF-36.

**Conclusions:**

The trans-oral endoscopic approach can acquire better cosmetic results and achieved high-level QOL.

## Introduction

After thyroidectomy, patients experience impaired quality of life (QOL). With the development of surgical techniques, endoscopic thyroid surgery has progressed and has been favored by more patients in China. These surgical techniques include the trans-axillary approach [1], the trans-anterior chest wall approach [2], the trans-areola approach [3], and so on. These surgical methods have avoided the formation of scars on the neck,but all still cause some scarring. Many young patients are concerned about the appearance of scars, especially young female patients worry about scars on the chest. Therefore, Trans-oral endoscopic thyroidectomy (ETVOA) has been developed as a new surgical technique via natural body orifices, which leaves absolutely no scar.. Furthermore, ETVOA is a safe and feasible surgical method [4, 5]. Compared with conventional open surgery, patients who receive ETVOA have higher satisfaction scores and a better QOL [6]. However, there is no relevant evidence indicating that the QOL of patients who undergo ETVOA is better than that of those who receive trans-areola endoscopic thyroidectomy (ETAA).We conducted this study to analyze the characteristics of patients who received ETVOA and ETAA and compare the differences in QOL with two endoscopic thyroidectomies.

## Materials and methods

### Inclusion/exclusion criteria

The study protocol was performed in accordance with the relevant guidelines and regulations. We reviewed patients who underwent ETAA and patients who received ETVOA from October 2018 to October 2019 in the First People’s Hospital of Zunyi. After the patient’s informed consent was received, they specified their preferred option. All patients routine laryngoscopy before thyroid operations. All of the surgeries were performed by two experienced laparoscopic surgeons. The inclusion criteria for patients eligible to participate in this study were as follows: (1) benign thyroid nodules such as thyroid cysts, single nodular goiter, or multinodular goiter; (2) 2 cm < tumor diameter < 5 cm; and (3) follicular tumor. The exclusion criteria were as follows: (1) Preoperative diagnosis of thyroiditis, Grave’s disease, toxic nodular goiters and other thyroid-related endocrine diseases; (2) pathologically confirmed thyroid cancer after surgery; (3) tumor diameter ≥ 5 cm; (4) history of neck surgery; and (5) converted technique from endoscopic approach to the open approach during surgery. A total of 131 patients were enrolled in this study, including 74 patients in the ETAA group and 57 patients in the ETVOA group. All of the patients were postoperatively diagnosed with benign thyroid disease.

### Surgical technique

#### ETAA

The main method of operation was hemithyroidectomy. Tracheal intubation was performed using general anesthesia. The patient was placed in a supine position with legs separated, head tilted back, and shoulders slightly elevated. (1) We made a horizontal incision of approximately 12 mm above the midpoint of the double-nipple connection 1.5 cm in front of the sternum skin. (2) Using vascular forceps, we separated the subcutaneous tissue up to the superficial layer of deep fascia. (3) After the subcutaneous tunnel was established, a 10-mm cannula (lens diameter, 10 mm) was inserted through the incision, and CO2 gas was injected to maintain a constant pressure of 6 mmHg. (4) We made a 5-mm incision on the left and right areola edges as the auxiliary operation hole and the main operation hole, and we inserted the lossless grasping forceps and the ultrasonic knife, respectively. (5) Under the direct view, we used the electrocoagulation knife to separate the deep surface of the cervical latissimus dorsi up to the thyroid cartilage, and we separated the front edge of the sternocleidomastoid muscle on both sides. (6) We cut the white line of the neck, and used the extracorporeal suspension line to retract the anterior cervical muscle group on the affected side, and cut the thyroid surgical capsule to reveal the thyroid tissue. (7) We cut the thyroid lobe with care taken to preserve the recurrent laryngeal nerve and the parathyroid. We removed the thyroid lobe. (8) Using white thread, we sutured the neck and subhyoid muscles; we guided a drainage tube with a side hole cut out from the contralateral areola incision. No antibiotics were used during the perioperative period. [7]

#### ETVOA

The main method of operation was hemithyroidectomy. The patient received general anesthesia after intubation. The primary surgical steps are as follows: (1) After the oral vestibule is exposed, we used iodophor and compound chlorhexidine to disinfect and rinse the oral cavity. (2) We injected the “expansion fluid” consisting of 500 mL of normal saline and 1 mg of epinephrine in the middle and lower portions of the oral vestibule. (3) We made a 12-mm incision in the middle of the oral vestibule and above the lower ligament and used the rod to separate along the lower layer of the platysma to the neck. (4) We established the surgical cavity and used a 5-mm trocar to insert into the observation hole. Subsequently, we injected CO2 gas to maintain pressure at 6 mmHg. (5) We cut both sides of the oral vestibular mucosa in 5-mm incisions and placed the corresponding trocar; the two holes are the main operation hole and the auxiliary operation hole. (6) Under direct vision, we used an ultrasonic knife to separate the subcutaneous, loose connective tissue of the mandible and neck to the upper sternum fossa, and the sternocleidomastoid on both sides of the muscle to establish a surgical space. (7) We used an ultrasonic knife to cut the white line of the neck and separate the anterior cervical muscles. An extracorporeal suspension wire was used to distract the subhyoid muscles and expose the thyroid gland. (8) We found the tumor and removed the thyroid lobe which containing the tumor. During the separation process, the energy surface of the ultrasonic blade was distant from the trachea, parathyroid gland, and recurrent laryngeal nerve. (9) The specimen sample was placed in the specimen bag and removed from the observation hole for pathological examination. The surgical wound was flushed using normal saline, and the wound was sutured after no active bleeding was detected. Antibiotics are used before and after surgery to prevent wound infection [8].

### Observed indicators and follow-up

The observed indicators include the patient’s gender, age, body mass index (BMI), tumor size, operation time, intraoperative blood loss, extubation time, length of hospitalization, hospital costs, and complications (RLN injury, Surgical site infection, Parathyroid injury, Hematoma). The patient’s postoperative follow-up includes Quality of Life Questionnaires that were used to assess the patient’s QOL at 2 weeks, 4 weeks, and 2 months after surgery. The specific questionnaires include 10 items [6]. Overall satisfaction and visual analogue scale (VAS) pain scores at the surgical site range from 0 to 10. Neck movement, shoulder movement, voice, and swallowing are evaluated based on subjective assessment: 0, never; 1, almost never (occasionally); 2, sometimes; 3, almost always; and 4, always. Tingling sensations and numbness in the neck and chin areas are evaluated according to the following: 0, no pain or other abnormal sensation; 1, minimum; 2, moderate; and 3, severe. Physical activity and psychosocial impairment are assessed by comparing the preoperative and postoperative status: 0, no damage; 1, almost never (occasionally); 2, sometimes; 3, almost always; and 4, always. A questionnaire was conducted to assess the patient’s general QOL using short-form health survey (SF-36). It includes eight parameters: physical function, role-physical, bodily pain, general health, vitality, social function, role-emotional, and mental health. The higher the score, the better the parameter. After the operation, patients would be followed up by the outpatient clinic or by telephone, and the questionnaire would be completed at the same time.

### Statistical methods

SPSS 19.0 (SPSS lnc. Chicago, IL, USA) was used to calculate and analyze the data. For quantitative data, we conducted a normality test. If the normal distribution is met, the data are expressed as the mean ± standard deviation values, and the t-test is used to analyze the data. If the distribution does not meet the normality, rank sum test is used. For classified data, a chi-squared test is used to analyze the data.

## Results

A total of 74 patients underwent ETAA, and 57 patients underwent ETVOA; the data from 131 patients were analyzed to compare the QOL outcomes between the two groups. We compared the clinical characteristics between the two groups and found that the clinical characteristics such as gender, age, BMI, and tumor size were similar between the two groups. In terms of intraoperative and postoperative complications, the two groups showed no statistically significant differences in the amount of intraoperative bleeding, injury to the recurrent laryngeal nerve (RLN), infection in the operating area, and hypoparathyroidism. There was also no difference in hospitalization costs between the two groups. The length of hospital stay and time for extubation of patients in the ETVOA group were significantly shorter (Table1).

**Table 1:** Patients’ characteristics

Characteristics	ETAA (n:74)	ETVOA (n:57)	*P*-Value
Gender: n (%)			
Male	26 (35.1)	21 (36.8)	*P* > 0.05
Female	48 (64.9)	36 (63.2)	
Age (years)	41.2 ± 11.9	37.8 ± 12.4	*P* > 0.05
BMI	23.9 ± 2.7	23.4 ± 2.9	*P* > 0.05
Tumor size (cm)	2.6 (3.15)	2.5 (2.95)	*P* > 0.05
Operative time (min)	104.9 ± 30.6	145.5 ± 33.6	*P* < 0.05*
Blood loss (milliliter)	32.2 ± 8.9	30.0 ± 8.8	*P* > 0.05
Extubation time (day)	4 (5)	0 (0)	*P* < 0.05*
Hospital stays (day)	5 (6)	3 (4)	*P* < 0.05*
Hospital costs (CNY)	12,823.5 (14,976.9)	12,159.68 (16,093.28)	*P* > 0.05
Complication: n (%)			*P* > 0.05
RLN injury	0	0	
Surgical site infection	3 (4.0)	1 (1.7)	*P* > 0.05
Parathyroid injury	2 (2.7)	1 (1.7)	*P* > 0.05
Hematoma: n (%)	1 (1.4)	1 (1.7)	*P* > 0.05

* *p* < 0.05: statistically significance

We evaluated postoperatively the patients’ QOL using two questionnaires (Table2). The follow-up time was at 2 weeks, 4 weeks, and 8 weeks. The ETVOA group performed better than the ETAA group in the following manner: there were significant decreases in neck numbness after surgery during the whole period, and there were significant decreases in neck movement impairment at 4 weeks after surgery. Furthermore, cosmetic and overall satisfaction were significantly better in the ETVOA group throughout the entire follow-up period.

**Table2:** Comparison of quality-of-life parameters between two group based on the specific thyroid surgery-related QOL questionnaire

Specific thyroid surgery-related QOL parameter and grade	Postoperative 2 weeks			Postoperative 4 weeks			Postoperative 8 weeks		
	ETVOA (n: 57)	ETAA (n:74)	*P*-value	ETVOA (n: 57)	ETAA (n:74)	*P*-value	ETVOA (n: 57)	ETAA (n:74)	*P*-value
Numbness N (%)									
0	24	16	0.044*	34	25	0.021*	42	32	0.005*
1	23	32		16	30		10	31	
2	7	18		6	13		5	10	
3	3	8		1	6		0	1	
Tingling N (%)									
0	9	13	0.969	16	20	0.959	27	36	0.804
1	20	24		25	33		21	23	
2	25	32		14	17		8	12	
3	3	5		2	4		1	3	
Cosmetic N (%)									
4	29	23	0.037*	34	25	0.013*	43	33	0.005*
3	17	20		15	23		10	26	
2	10	29		8	25		4	14	
1	1	2		0	1		0	1	
0	0	0		0	0		0	0	
Voice impairment N (%)									
0	48	58	0.687	52	66	0.870	55	68	0.276
1	7	13		4	7		2	6	
2	2	3		1	1		0	0	
3	0	0		0	0		0	0	
4	0	0		0	0		0	0	
Swallowing impairment N (%)									
0	42	48	0.701	48	53	0.388	52	60	0.421
1	6	9		4	11		3	7	
2	7	12		3	6		1	4	
3	2	5		2	4		1	3	
4	0	0		0	0		0	0	
Neck movement impairment N (%)									
0	11	17	0.854	25	15	0.041*	33	35	0.762
1	10	16		15	20		13	18	
2	14	19		8	18		6	12	
3	7	8		4	11		3	5	
4	15	14		5	10		2	4	
Shoulder movement impairment N (%)									
0	49	64	0.651	52	66	0.699	54	69	0.723
1	8	9		5	8		3	5	
2	0	1		0	0		0	0	
3	0	0		0	0		0	0	
4	0	0		0	0		0	0	
Physical activity reduction N (%)									
0	35	40	0.881	45	51	0.574	51	63	0.431
1	7	11		8	13		6	9	
2	5	10		3	7		0	2	
3	6	7		1	3		0	0	
4	4	6		0	0		0	0	
Psychosocial impairment N (%) Psychosocial impairment N (%)									
0	44	54	0.649	50	62	0.574	55	69	0.589
1	7	11		6	7		2	4	
2	4	6		1	4		0	1	
3	2	1		0	1		0	0	
4	0	2		0	0		0	0	
VAS Pain score (0–10) Mean (SD)	3.1 (1.5)	2.9 (1.6)	0.479	2.2 (1.3)	1.9 (0.8)	0.088	1.3 (1.1)	1.2 (0.9)	0.156
Overall satisfaction (10–0) Mean (SD)	9.2 (1.3)	8.9 (1.5)	0.042*	9.5 (0.9)	9.1 (1.5)	0.035*	9.8 (0.7)	9.3 (1.1)	0.023*

* *p* < 0.05: statistically significance

The SF-36 questionnaire demonstrated no significant difference in the scores of physical-related parameters between the two groups; however, in Social function and Role emotion due to patients’ emotional problems and mental health. Social function scores were significantly higher in the ETVOA group compared to the ETAA group at 2 weeks and 4 weeks after surgery. The score of role emotion caused by emotional problems in the ETVOA group is higher than that in the ETAA group at 2 weeks and 4 weeks after surgery. The score of mental health in the ETVOA group is higher than that in the ETAA group, but this occurred only at 4 weeks after surgery. The difference in other parameters between the two groups was not observed to be statistically significant. (Table3).

**Table 3:** Comparison of quality-of-life parameters between two group based on SF-36 questionnaire

SF36	Postoperative 2 weeks			Postoperative 4 weeks			Postoperative 8 weeks		
	ETVOA (n: 57)	ETAA (n:74)	*P*-value	ETVOA (n: 57)	ETAA (n:74)	*P*-value	ETVOA (n: 57)	ETAA (n: 74)	*P*-value
Physical function	85.1 (20.6)	80.1 (21.3)	0.365	94.2 (23.5)	87.6 (21.8)	0.107	96.4 (23.6)	91.4 (20.3)	0.364
Role physic	84.2 (18.4)	70.1 (26.4)	0.175	87.5 (28.9)	78.3 (33.1)		98.3 (17.6)	88.9 (26.8)	0.235
Bodily pain	77.8 (23.1)	64.3 (40.2)	0.148	89.4 (23.4)	79.1 (26.9)	0.279	93.1 (19.5)	86.7 (24.5)	0.541
General health	73.5 (29.4)	69.8 (31.2)	0.962	82.1 (27.3)	74.6 (22.5)	0.319	85.4 (18.9)	83.3 (25.8)	0.251
Vitality	66.7 (27.8)	60.6 (26.2)	0.073	77.5 (21.7)	71.8 (26.3)	0.372	81.1 (25.6)	75.9 (32.3)	0.557
Social function	80.3 (20.3)	61.8 (25.9)	0.035*	88.9 (23.8)	72.5 (20.4)	0.017*	91.2 (18.2)	88.6 (33.5)	0.068
Role emtion	86.4 (19.7)	67.8 (28.4)	0.028*	91.6 (18.6)	81.3 (25.8)	0.031*	92.2 (16.5)	91.7 (17.9)	0.981
Mental health	76.5 (24.5)	70.2 (26.9)	0.076	89.8 (19.7)	80.8 (22.8)	0.027*	89.2 (23.3)	91.6 (16.5)	0.875

* *p* < 0.05: statistically significance

## Discussion

In 1997, Dr. Huscher completed the first endoscopic thyroid gland lobectomy [9]. At present, various approaches to endoscopic thyroid surgery are widely performed. With the continuous development of medical technology and improved cosmetic surgery techniques, thyroidectomy via the oral approach came into being. In 2009, the German physician Wilhelm completed endoscopic thyroid surgery via the floor of the mouth for the first time [10]. Japanese scholar Nakajo [11] and Chinese scholar Wang [12]took the lead in reporting that ETVOA was completely scar-free compared with breast approach, axillary approach, and areola approach. In addition to scars, the patients’ QOL after surgery is an important indicator for evaluating the type of surgery and the surgical outcome. We compared the patients’ QOL using the ETVOA approach and ETAA approach. The results showed that the cosmetic effect of the ETVOA approach is satisfactory. It is more beneficial for patients to feel self-confidence after surgery, be able to rapid recovery, and improve their QOL.

In this study, the clinical characteristics of the patients showed that there were no differences in gender, age, BMI, tumor size, intraoperative blood loss, and hospitalization costs. There was a significant difference in the operation time between the two approaches. Compared with the ETAA approach, the operation time for the ETVOA approach is longer, as noted in previous studies [13]. We speculate that the main reasons may be due to the following two aspects: (1) The operation space using the ETVOA approach is relatively narrower than the operation space using the ETAA approach; and (2) the surgeon’s proficiency at the time when the ETVOA was performed in the early stage is consistent with the findings shown in previous studies [14]. Previous study showed that better surgical operation and experience can reduce the necessary operation time [15]. In addition, compared with the conventional placement of drainage tubes in the ETAA approach, there is no need to place drainage tubes in the ETVOA approach. In both groups, there was no difference in the incidence of hematoma formation in the operation area after surgery (Table1). Therefore, there is a significant difference in drainage volume between the two groups. Before the patient is discharged, the drainage tube is routinely removed, so the use of drainage tubes will increase the length of hospitalization for patients undergoing ETAA surgery [16]. All patients used painkiller (non-steroidal analgesic) for 2 days analgesic, while only the ETVOA group used antibiotics for 2 days. In the observation indicators, there was no difference in postoperative infections in the both groups. Although the transoral approach may cause the incision to become contaminated, but the reasonable use of antibiotics, the probability of infection will become very low. In the two groups of patients, we did not find significant differences in pain scores and infection rates. In our study, only 3 patients had parathyroid injury (Table1).The reason may be due to the heat of the ultrasonic knife during the operation. For patients with hypocalcemia caused by parathyroid injury during surgery, we routinely give oral calcium or vitamin D.

In terms of QOL, the patients in the two groups had different degrees of discomfort after surgery. The main complaint was numbness of the neck, which was caused by damage to the skin nerve when the approach was established during surgery. The numbness of the oral approach is mainly the area between the lower jaw and the lower lip, and the areola numbness is the area from the areola to the thyroid cartilage. The incidence of neck numbness was different between the two groups. The number of patients with neck numbness in the ETVOA group was significantly less than that in the ETAA group throughout the entire follow-up period. The reasons are that the tunnel through the areola approach is longer, the separated skin area is larger, the damaged skin area is larger, and the likelihood of skin nerve damage is higher. In addition, all of the patients had a tingling sensation in the surgical area; however, our research outcomes demonstrated that there was no significant difference in the tingling sensation between the two groups. At the same time, we observed that the patients in the ETAA group had greater postoperative neck movement impairment than those in the ETVOA group, although this situation occurred only in the fourth week. We speculated that using the ETAA approach caused greater anatomical damage to the human body and more scarring. With the patient’s rehabilitation exercise and scar softening, there was no significant difference in neck activity between the two groups after 8 weeks of surgery [17]. It has been suggested that the formation of neck scars may affect the patient’s voice and swallowing [18]. Our results indicated that the patients in both groups exhibited no differences in voice and swallowing impairment at 2 weeks, 4 weeks, and 8 weeks after surgery. This finding indicated that the patient’s postoperative dysphonia and dysphagia are not due to the formation of neck scars, and that the patient’s transient postoperative dysphonia may be due to edema in the postoperative area. In addition, the two indicators of shoulder movement impairment and physical activity reduction were not significantly different between the two groups. This outcome is consistent with the role emotion in the SF-36 questionnaire. The postoperative VAS pain score and the bodily pain in the SF-36 questionnaire did not differ between the two groups, which is due to the routine use of analgesic agents after surgery and the gradual relief of pain after surgery.

The patients’ cosmetic satisfaction in the ETVOA group was significantly better than that in the ETAA group, which corresponds to the relevant indicators in the SF-36 scale. Patients who underwent ETVOA surgery have higher cosmetic satisfaction, better social function and fewer mental health problems, which helps patients quickly return to normal life and work. Therefore, the overall satisfaction of the patients is higher, and the patients will experience a high-level QOL. The scores of role emotion caused by social function and emotional problems in the ETVOA group were higher at 2 weeks and 4 weeks after surgery, which may be likely attributable to the visible scars on the body surface and the wider scar area in the surgical area. These findings also confirm that the ETVOA approach is superior to the ETAA group. General health and vitality are two important indicators for assessing patients’ QOL; however, there is no difference between the two groups. In general, the QOL benefit was greater in the ETVOA group than that in the ETAA group.

To the best of our knowledge, this is the first study to compare the effects of ETVOA endoscopic thyroidectomy and ETAA endoscopic thyroidectomy with regard to general and specific health aspects of patients. Better QOL results were observed in the ETVOA group. However, this study has several limitations, including the small sample size and short follow-up time. At present, only a very small number of patients with malignant thyroid disorders can be operated using the ETVOA approach. Therefore, the QOL results can only reflect the satisfaction of patients with benign thyroid mass. With the continuous innovation of endoscopic technology, the feasibility and the safety of trans-oral thyroid cancer surgery will be improved. Furthermore, the ETVOA approach exposes the surgical field of the lower thyroid and the seven areas of the neck better [19]. For some patients with tumors in the middle and lower lobes of the thyroid gland and for patients with suspected cervical lymph node metastasis in the 7th region of the neck before surgery, the ETVOA approach can provide enhanced surgical vision [20]. Therefore, the ETVOA approach is an excellent choice for treating patients with certain types of malignant thyroid tumors.

## Conclusions

The two types of endoscopic thyroid surgery are safe treatment for patients with benign thyroid nodules that require surgical treatment. The oral vestibular approach can acquire better cosmetic results and make patients quick return to normal life. Therefore, ETVOA is worthy of being further promoted and applicated for patients with benign thyroid nodules that require surgery.

## Data Availability

The datasets used and/or analyzed during the current study are available from the corresponding author on reasonable request.

## Abbreviations

ETVOA: Endoscopic thyroidectomy via the oral vestibular approach
ETAA: Endoscopic thyroidectomy via the areola approach
QOL: Quality of life
SF-36: The 36-Item Short Form Survey
BMI: Body mass index
VAS: Visual analogue scale
RLN: Recurrent laryngeal nerve
